# Assessing augmented reality displays in laparoscopic liver surgery - a clinical experience

**DOI:** 10.1007/s00464-025-12008-5

**Published:** 2025-07-25

**Authors:** João Ramalhinho, Sandun Bulathsinhala, Kurinchi Gurusamy, Brian R. Davidson, Matthew J. Clarkson

**Affiliations:** 1 UCL Hawkes Institute, Department of Medical Physics and Biomedical Engineering, UCL, London, UK; 2 Division of Surgery and Interventional Science, UCL, London, UK

**Keywords:** Laparoscopy, Liver surgery, Augmented reality, Visualisation

## Abstract

**Purpose:**

Augmented Reality (AR) is considered as a potential image guidance tool to increase the safety of laparoscopic liver surgery. By overlaying surface models of the liver and hepatic blood vessels derived from pre-operative 3D imaging onto the laparoscopic video images, surgeons can have more context on the surgical scene and perform more informed decisions. Although several techniques to enable AR have been reported, few studies have addressed clinical deployment feasibility and visual display requirements.

**Methods:**

We present the results of a prospective study where a previously reported AR system, the Smartliver, has been evaluated with 13 participants consented for laparoscopic liver surgery. The system is optically tracked, the laparoscope is pre-operatively calibrated, and the AR overlay is obtained manually during surgery. We have prospectively tested 3 different AR displays and have obtained feedback from surgeons through Likert Scale questionnaires.

**Results:**

Questionnaire results on the suitability of displays for surgical use suggest the Smartliver AR overlay is intuitive, can help on decision-making, and has potential for future use. Surgeons showed preference for a display where only a restricted circular “hole” region of the 3D model is shown.

**Conclusion:**

Optically tracked AR is easy to deploy, and surgeons agree on its potential to improve surgical outcomes of laparoscopic liver surgery. Future developments should focus on automating the AR overlay alignment process, predicting liver surface intra-operative deformations, and optimising the optical tracking system positioning

Liver cancer is a major health problem ranking as the fifth most deadly neoplasm at a global scale [1]. For most primary and metastatic liver cancers, surgical resection of these tumours is usually the preferred treatment option [2]. Compared to an open approach, laparoscopic liver resection offers well-established benefits in terms of reduced trauma to the patient, shorter hospital stays, and reduced costs to the hospitals [3]. However, patients with tumours that are either close to major blood vessels or located posteriorly are deemed too high a risk for laparoscopic resection [2]. To enable safer laparoscopic surgery and increase its uptake, Augmented Reality (AR) has been proposed as an image guidance technology that allows surgeons to perceive a semi-transparent overlay of the liver surface and corresponding internal anatomy onto the laparoscopic camera video [4]. This overlay information is usually extracted from a segmented 3D model produced from the individual patient pre-operative contrast CT.

Most technical developments for AR in laparoscopic liver surgery have been directed towards the problem of registering, i.e. aligning the pre-operative model of an individual’s liver from CT on the laparoscopic video findings to produce an accurate overlay [5]. Although there are several improvements in registration automation [6, 7], prediction of intra-operative liver deformations [8], and depth perception [9, 10], few studies have addressed the usability and ease of deployment of laparoscopic liver surgery AR systems in the clinical setting. Using mockup surgical setups, previous studies have reported how AR can help surgeons localising structures below the liver surface more accurately [11–13]. On live surgery, studies have focused on assessing registration accuracy, both when acquired automatically [14] or semi-automatically [15]. Overall, all studies have focused mainly on the quantification of accuracy, but not on other system design considerations such as the AR display method.

In this paper, we present results from a prospective feasibility study using a previously developed AR system, the Smartliver system [12, 15, 16] on a cohort of 13 participants undergoing laparoscopic liver surgery. Compared to previous works, the aim of this study was to verify the feasibility of using these systems and understanding what are the requirements for an optimal display of AR on the operating room. This paper presents the following contributions:

- The first study to compare multiple display options for AR during live laparoscopic liver surgery.
- A set of conclusions to further inform future development of AR for laparoscopic surgery.

## Methods

In this study, we have prospectively tested different AR displays with the Smartliver system [12] on 13 laparoscopic liver surgery procedures, and feedback was obtained through questionnaires. The next subsections detail all relevant study components.

### Participant data

Data was acquired during 13 laparoscopic liver surgery procedures performed between November 2023 and September 2024 at the Royal Free Hospital in London, UK. Data collection during this prospective study was approved by a local research ethics committee (Reference: 14/LO/1264 & 10/HO720/87) and registered with ISRCTN (ID: 77923416). Recruited patients provided written consent to participate. During the study, the Smartliver AR displays were not used to guide the surgical procedure. Instead, ease of deployment and usability aspects on visualisation were evaluated. A summary of the 13 consented participants is presented in Table 1. Each row shows whether laparoscopic resection or diagnostic laparoscopy was performed, the indication, and version of AR Display that was prospectively tested (Displays are explained in subsection 2.3 below). Except for participants 8 and 13, interaction with the AR system was always performed by a different surgeon. Of the 12 considered surgeons, 2 have reported being previously exposed to AR in live laparoscopy (participants 6, 8, 13) and 3 have reported experience with AR in a training setting (3, 7, 11). Since the aim of this study was not to assess the effect of AR on surgical outcomes of laparoscopic liver resection, patients consented for abdominal diagnostic laparoscopy were considered as well.

**Table 1:** List of consented participants included in this study

	Procedure	Indication	AR Version
1	Resection	Resection of hepatocellular carcinoma on left lobe	Display 1
2	Resection	Resection for gallbladder carcinoma	Display 1
3	Diagnostic	Staging for duodenal adenocarcinoma	Display 1
4	Resection	Resection of colorectal metastasis on left lobe	Display 1
5	Diagnostic	Staging for pancreatic head adenocarcinoma	Display 1
6	Diagnostic	Staging for intraductal papillary carcinoma suspicion	Display 1
7	Diagnostic	Staging for pancreatic ampullary carcinoma	Display 2
8$^{*}$	Diagnostic	Staging for pancreatic head adenocarcinoma	Display 2
9	Diagnostic	Staging for pancreatic ampullary carcinoma	Display 2
10	Diagnostic	Staging for hilar cholangiocarcinoma	Display 3
11	Diagnostic	Staging for pancreatic head adenocarcinoma	Display 3
12	Resection	Resection of multiple left lobe adenomas	Display 3
13$^{*}$	Diagnostic	Staging for hilar cholangiocarcinoma	Display 3

For each of the 13 rows we present the surgical procedure, indication, and displayed AR version. In the procedure column, diagnostic refers to diagnostic laparoscopy and resection refers to liver laparoscopic liver resection. A * highlights two participants where responses were provided by the same surgeon

### SmartLiver AR system

The Smartliver system is an optically tracked AR system with a Video-see Through (VST) display designed for laparoscopic liver surgery. In terms of hardware, this system consists of a medical grade computer that can be attached to any laparoscopic system stack, and an optical tracking [17] system. In the present study, we have used a Storz^1^ 3D laparoscope and an NDI Polaris Spectra^2^ for tracking.

**Fig. 1:**
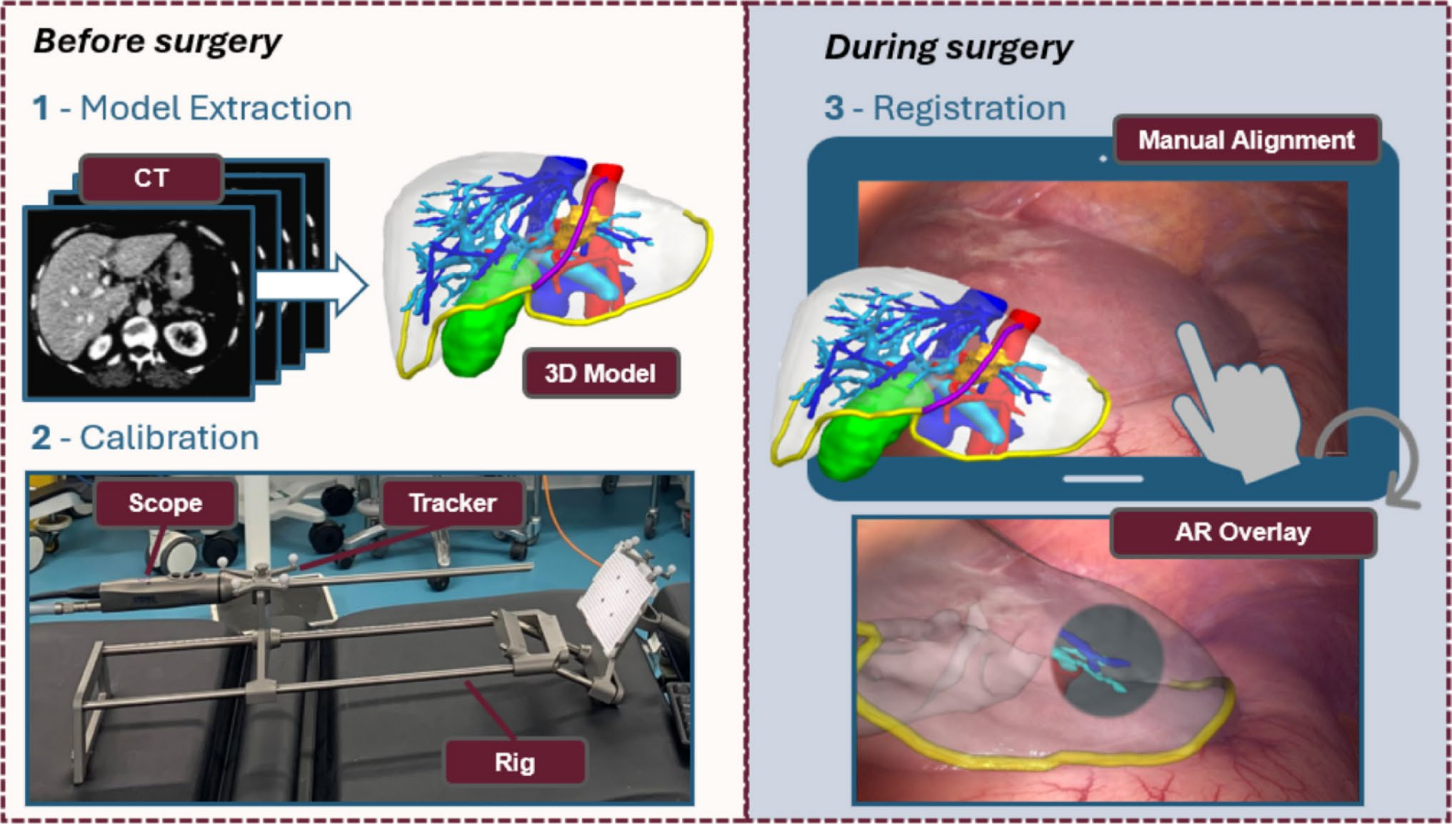
Key deployment steps for the Smartliver AR system. Steps on the left are performed pre-operatively, whereas the manual registration is performed live. The clinical example shown in this figure refers to Participant 13 of the present study, where Display 3 was used

In terms of software, the Smartliver system has a dedicated Graphical User Interface (GUI) to display AR separately from the main laparoscopic system screen. To enable AR during surgery, the system requires three key steps illustrated in Fig. 1. Before surgery, 3D models of the liver, hepatic vessels, gallbladder, and tumours are extracted from the CT of the patient using a commercial service^3^ (step 1). In order to enable the live update of the AR overlay given any position of the scope, a calibration process is needed to map the geometric relationship between the laparoscope camera and the optical tracking system attached to the stack. This is achieved by placing a tracking collar close to the handle of the scope, and performing the rig-based calibration method reported in [18] (step 2). During surgery, the Smartliver GUI is used to load the 3D model of the liver, and manual mouse controls are used to align it with a fixed view of interest of the liver (step 3). After completing this step, surgeons can observe the AR display updating with the motion of the tracked laparoscope.

### Evaluation protocol

We have followed the Smartliver workflow of Fig. 1 for each of the 13 consented participants. To ease deployment and minimise disruptions to the clinical workflow, the calibration of the laparoscope was performed a single time pre-operatively, and the resulting calibration transformations were applied in for all participants. For this to be possible, the tracking collar was attached and aligned with the base of the laparoscope in the same configuration at which the laparoscope was calibrated. The manual registration step was completed by two engineers: one engineer used the system for participants 2 and 13, the other one for the remaining 11. Manual registration was always conducted at the beginning of the procedure - this was necessary to not influence surgical decision-making on the remaining time of the surgery. During this step, the operating surgeon provided feedback on the geometrical correctness of the alignment.

#### AR displays

**Fig. 2:**
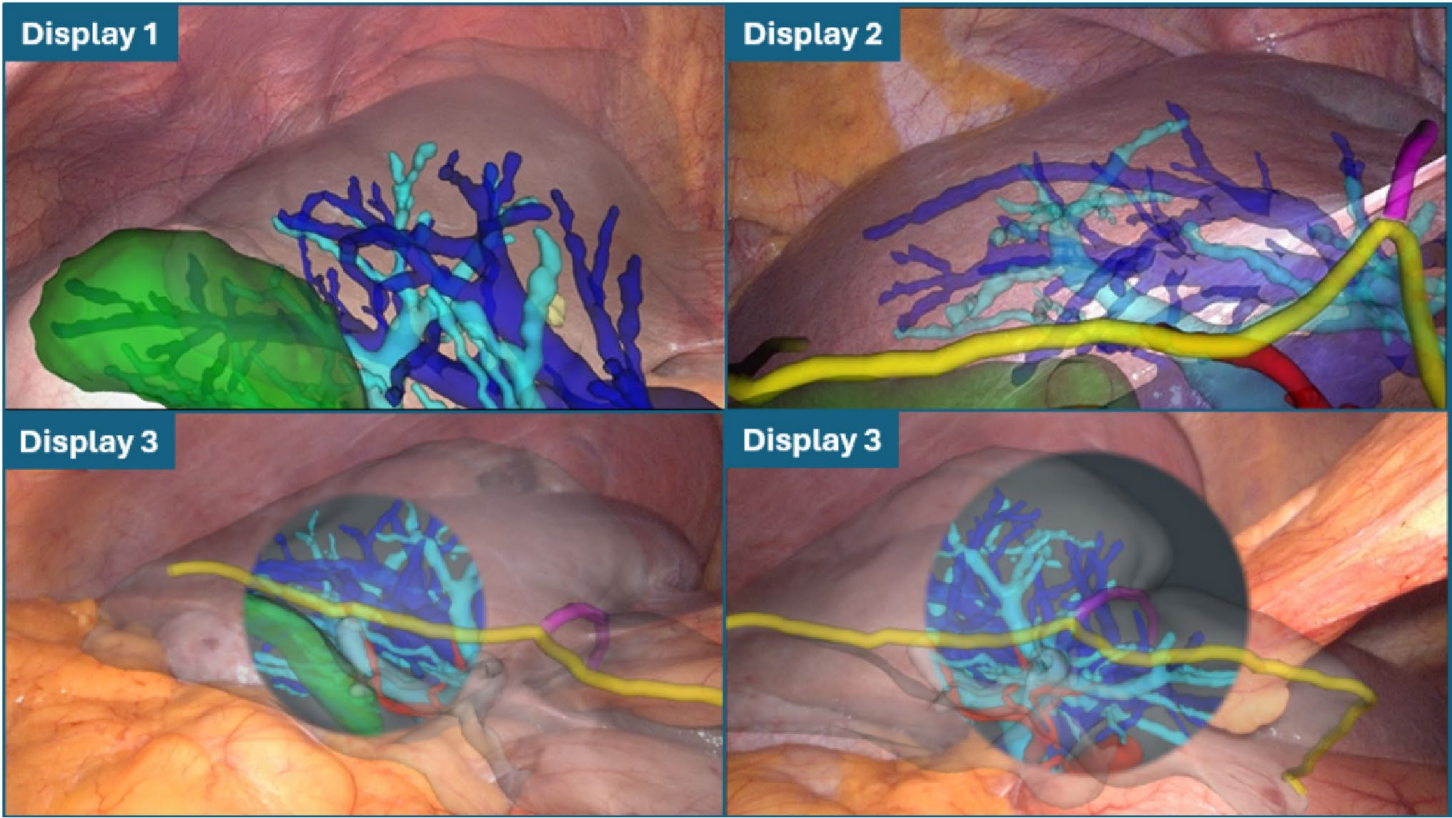
Set of tested AR displays on a selection of participants. Top left image shows Display 1, the conventional AR display. Top right image shows Display 2, where the anterior ridge and ligament are shown as well. The two bottom images show Display 3 - left with the medium size option and the right with large size option. Across all displays, light blue represents Portal Vein, blue the Hepatic Veins, red the hepatic arteries, green the gallbladder, yellow the anterior liver ridge, purple the falciform ligament, and white the liver surface. Left top image was acquired from Participant 5, top right acquired from Participant 7, and the two bottom images acquired from Participant 10. Bottom left image shows the medium size circle of Display 3, whereas the bottom right shows the large size

As listed in Table 1, we have prospectively tested three different displays throughout this study. The aspect of each of these displays is illustrated in Fig. 2. For the first 6 patients, we have used Display 1 which consists of rendering all internal structures of the liver and the liver surface. To enable visibility of the internal anatomy, the transparency of the liver surface is increased. After realising in these first participants that it is challenging to intuitively understand the 3D orientation and position of a transparent liver surface, we have introduced Display 2. In this display, we show two additional liver contour cues that allow for a better visual match between video and surface model - the anterior ridge of the liver (yellow) and falciform ligament (purple). Both contours were manually extracted from the 3D liver surface model before surgery.

On the last four participants, we have introduced an alternative display option to reduce the perception of “clutter”, i.e. the amount of unnecessary graphical information in the view. As illustrated in the bottom row of Fig. 2, Display 3 only shows the internal structures of the 3D model only inside a circle centred on the laparoscopic video image. This visualisation allows the surgeon to point the scope to a region of interest and perceive the overlay as “hole” that can be peered through to inspect the internal anatomy. The size of this circle can be adjusted to three diameters that are set as a percentage of the video image height: small - 40%; medium - 60%; and large - 80%. Visibility of non-internal structures (liver and contour cues) are maintained to still provide context on the complete overlay.

#### Data collection

**Fig. 3:**
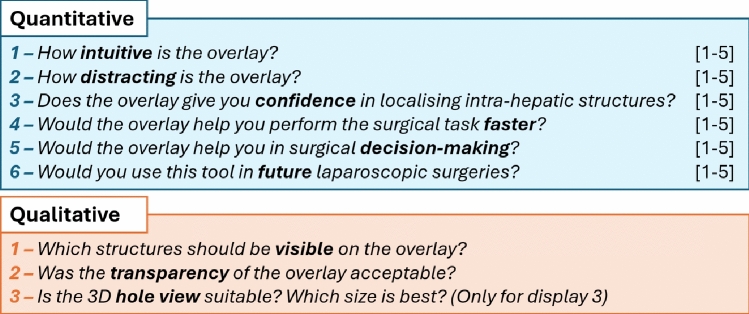
Set of questions asked to surgeons about the AR display. Blue highlights questions answered in Likert scale (0–5) and orange highlights questions open for feedback. For Quantitative question 2, we consider 5 as a not distracting overlay

Once an AR display was provided, the operating surgeon was allowed to interact with the overlay by moving the laparoscope. To evaluate the tested display, the surgeon was then asked to provide responses to a questionnaire with questions listed in Fig. 3, while observing the overlay. The first set of questions was answered on a Likert scale of 1–5 (1 - highly disagree, 5 - highly agree), whereas the last three questions were open for qualitative feedback. For question 2, the scale is inverted so that higher scores indicate less distraction. For the second participant, we were not able to record a response for question 6. The last qualitative question regarding the hole view was only asked when Display 3 was used.

## Results

### System setup

**Fig. 4:**
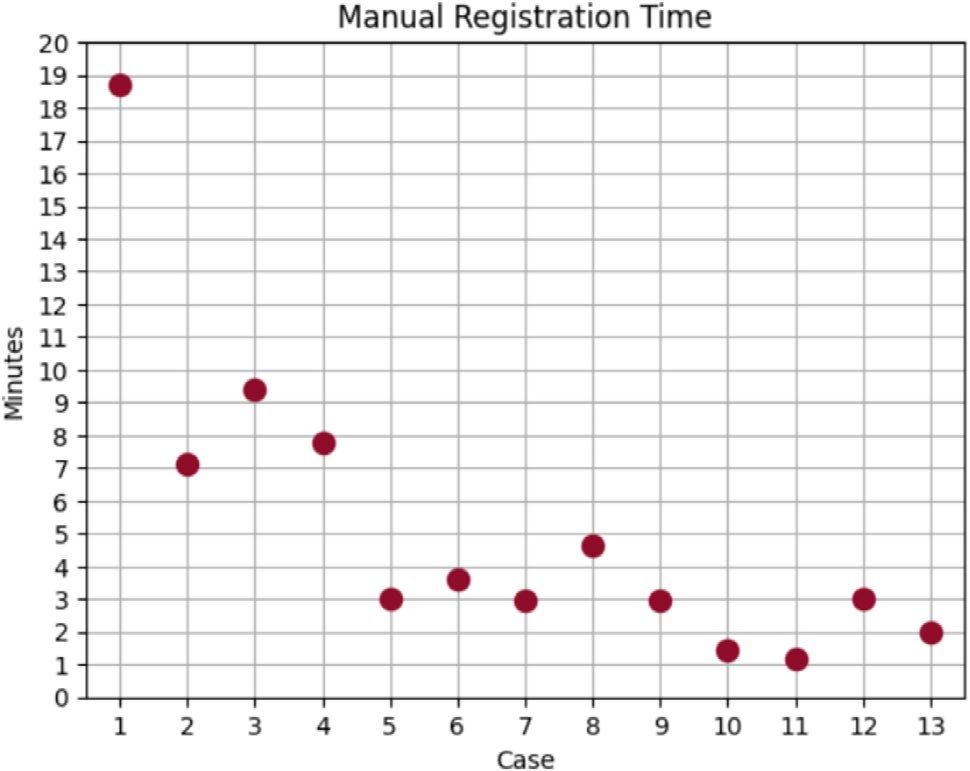
Time spent in minutes for engineers to achieve manual registration for each of the 13 participants

In addition to the questionnaire feedback, we measured the time taken to setup the different components of the AR system. These include the time taken to adjust the tracking collar position on the laparoscope and to perform the manual registration using the Smartliver GUI. This step was undertaken by the engineer performing the manual alignment and the scrub nurse present at the surgery. We have observed that the tracking collar attachment was consistently done in less than one minute before the laparoscope was inserted in the patient. We show the manual registration time taken in minutes for each participant in Fig. 4. We can observe that for the first 4 participants, times above 7 min were taken to perform alignment, whereas the last 5 participants never surpassed 3 min. This data suggests that there is a learning curve in this process and that integration of the AR system can be successively faster given appropriate training.

### Quantitative feedback

Likert scale results for the first set of questions versus the used AR displays are presented in Table 2. For each display and question, we present the median and Interquartile Range (IQR) across the number of participants. Overall, surgeons agree that the AR overlay has potential future use and can improve surgical decision-making. Between the three different displays, surgeons suggest that the hole-view (Display 3) is the most intuitive (score of 4.00 (0.75)) and leads to highest confidence in localising structures (score of 4.00 (0.00). Surgeons perceived Display 1 as the least distracting (score of 4.00 (2.00)), and did not have a strong opinion on whether any AR display could help speeding up the surgical procedure (score of 3.00 (1.00)).

**Table 2:** Quantitative feedback results obtained through the questions listed in Fig. 3

Questions	All	Display 1	Display 2	Display 3
Intuitive	4.00 (1.00)	3.50 (2.25)	3.00 (2.00)	${\textbf {4.00}}~(0.75)$
Distracting	${\textbf {4.00}}~(1.50)$	4.00 (2.00)	3.00 (3.00)	3.50 (1.75)
Confidence	4.00 (1.00)	3.50 (2.50)	3.00 (1.00)	${\textbf {4.00}}~(0.00)$
Faster	3.00 (1.00)	3.0 (1.25)	${\textbf {4.00}}~(2.00)$	3.00 (1.5)
Decision-making	4.00 (0.50)	4.00 (1.50)	4.00 (3.00)	4.00 (0.75)
Future use	4.00 (1.00)	${\textbf {5.00}}~(1.50)$	4.00 (2.00)	4.00 (0.75)

Bold values highlight the maximum median values obtained for each question

For each question and display, we present the Median and IQR across the number of participants. The column “All” refers to all 13 participants. Note that Future Use results for Display 1 have one missing value

**Fig. 5:**
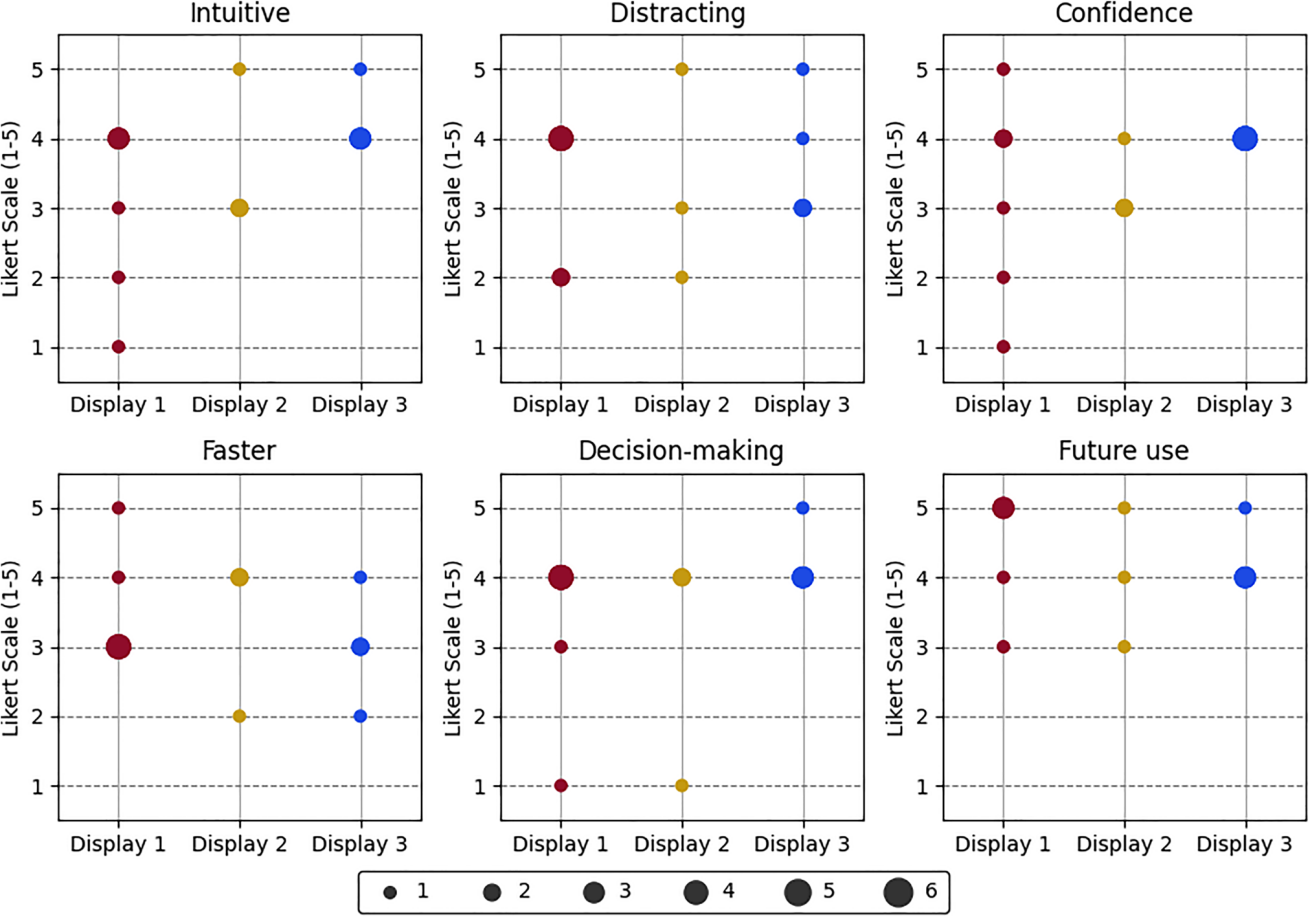
Questionnaire results for each of the six questions for each of the three displays. Red shows Display 1, Yellow shows 2, Blue shows 3. For each display, marker size at each score is scaled according to the number of surgeons that reported that score, as described by the horizontal bar at the bottom of the charts. As an example, the Confidence score for Display 3 was 4 for all surgeons, leading to a single, larger marker. Since there is a missing response on the future use question for the second participant, the total frequency for Display 1 in this case amounts only to 5

Since we have a small sample for each of the displays, we further present our Likert results in the charts of Fig. 5 for a more detailed analysis. In each chart, each dot marker represents the frequency of each Likert scale score (1-5) with its size. For Display 1 (red markers, 6 participants), it is clear that lower scores below 3 are present in all categories except for the“Future Use” and “Faster” questions. Display 2 (yellow markers, 3 participants) also shows lower values, but with fewer samples. Highest values were consistently obtained for Display 3 (blue dots, 4 participants), with only a score below 3 for the “Faster” question.

### Qualitative feedback

#### Structure visibility

 There was no consensus among surgeons on which structures of the AR overlay should be visible during the procedure. For Display 1, 4 out of 6 surgeons agreed that the liver surface should be present and visible, while the remaining 2 preferred to focus only on internal anatomy. 

For Display 2, 2 out of 3 surgeons agreed the liver surface should be visible, but one further elaborated that the liver display is mostly useful to assess the alignment of the overlay. The remaining surgeon suggested that the gallbladder should not be visible due to excessive clutter in the view. The 3 surgeons also agreed that the falciform ligament and ridge features should always be visible as well.

Only 2 surgeons gave feedback on structure visibility on Display 3, both agreeing that the contour features information is more valuable than the liver surface itself.

#### Display transparency

 Transparency of different structures was adjusted prospectively throughout the study. During the deployment of Display 1, the first 3 surgeons considered the liver surface transparency as too low to properly see the video image underneath. After adjusting liver transparency in Display 1, 1 surgeon suggested that blood vessel transparency should be reduced as well. The 3 surgeons that experienced Display 2 with increased blood vessel and liver surface transparency still considered multiple features to not be transparent enough. The last 4 surgeons testing Display 3 found all structure transparency levels to be acceptable.

#### Hole size

 For the participants using Display 3, there was no consensus on which hole size was optimal. One surgeon suggested this should dependent on the task, two suggested it should be the largest size and the remaining one preferred the smallest.

## Discussion

The results of this prospective study suggest the Smartliver AR system is a tool which surgeons feel could improve the outcome of laparoscopic liver surgery. The system is easy to deploy and does not present any substantial interruption to the clinical workflow. The only time-consuming step was the manual alignment, which can be performed under 3 min after appropriate training (Fig. 4) or automated with existing technology [6, 7]. For all AR options, there was a consensus on the potential of this AR tool to help on surgical decision-making. There was controversy on whether the AR system is likely to result in faster surgery as the interpretation of the AR display was felt to require time to assess during the surgical procedure.

The obtained quantitative scores indicate that the most suitable option for AR display is the hole-view of Display 3. This option was considered to be the most intuitive and to provide more confidence in structure localisation. This can be explained by the reduced clutter of the view - in this visualisation option, the internal anatomy overlay is narrowed to a smaller region that does not show the video as background. This overlay of anatomy on a black background may explain why surgeons testing this option found the structure transparency to be always acceptable. Display 2 results did not show substantial improvements over Display 1 (Table 2). However, the surgeons testing this display mentioned that the inclusion of contour features was useful to understand the alignment of the overlay.

Feedback on the structure visibility suggests that surgeons can have different requirements for AR visualisation. Although all surgeons agreed the internal anatomy and contour features (in Display 2 and 3) should be always visible, there was no consensus on whether the liver surface should be visible or not. Therefore, the option to switch the visibility of different structures should be selected based on surgeon preferences. There was no consensus on the optimal level of structure transparency as well - however, when surgeons experienced the display that seemed most intuitive (Display 3), transparency was always deemed as acceptable. Regardless, this parameter should be systematically tested in further evaluations. For the hole size of Display 3, it is clear that there is no optimal size. Depending on the anatomy and proximity of the scope to the liver, a fixed hole size can provide very different contexts.

### Study limitations

Limitations of our study include the lack of a direct comparison between displays for each participant and the lack of post-operative data to assess the impact of the AR displays on surgical outcomes. Conducting a comparison such as the one in [12] would be very difficult due to prospective nature of this study - our different display methods were developed during the course of the study. Additionally, such comparison would require more disruption to the workflow as an extra task would have been required to objectively compare each display. Such task would be likely to influence the perception of the surgeon and therefore the remnant of the surgery, transgressing the study ethics. Nonetheless, we intend to include this objective analysis in a future study.

Post-operative data was not reported in this study as the AR system was not used with the purpose of guiding the surgical procedure. The preliminary results here obtained are meant to inform a future study where surgical outcomes and clinical benefit of our AR system will be evaluated.

### Future recommendations

From our experience in this study, there are three key aspects that require improvement for further deployment. Firstly, although it is feasible to rapidly perform the manual alignment, the process should be further automated [6, 7] so that it can be repeated in multiple views, whenever needed during the course of surgery. Secondly, this system does not account for the intra-operative deformation that the liver experiences due to the abdominal cavity insufflation, i.e. pneumoperitoneum. By aligning surfaces from a non-deformed CT surface to the deformed video, the AR overlay can look particularly inaccurate on some views. We aim to improve this aspect by integrating automated deformable registration algorithms once the manual or automated alignment is completed [19]. Thirdly, maintaining a clear line of sight between the optical tracking camera and the tracking collar was a challenge in some surgical procedures. Depending on the overall positioning of the patient and tracking camera, the movement of the scope can break this line of sight between tracking collar and camera, interrupting the AR display. We intend to attach the optical camera in a configuration that is more flexible and has more range, increasing adaptability to different patient positions and laparoscope motions.

## Conclusion

We have prospectively evaluated an AR system for laparoscopic liver surgery on a cohort of 13 patients. Surgeons agreed they would use the system in the future, and that it could improve surgical decision-making. We have shown three different AR display methods, and found that restricting the internal anatomy visualisation to a smaller circle improves the overall usability of the system. Improvements are still required to automate the alignment, increase the realism of the overlay with intra-operative deformations, and to maintain the line of sight between optical camera and tracking collar during surgery.

## Footnotes

1 (http://www.storz.com).

2 (http://www.ndigital.com).

3 www.visiblepatient.com.
